# Virtual Reality Induced Awe in Chronic Low Back Pain: A Mixed-Methods Study

**DOI:** 10.3390/bs16091524

**Published:** 2026-08-29

**Authors:** Emma R. Tillery, Lydia C. Kang, Misha Shah, Arthur A. Raney, Andrea Stevenson Won, Valentina Mancuso, Mark Vorensky, Corey Shum, Zina Trost

**Affiliations:** 1 Department of Psychological and Brain Sciences, Texas A&M University, College Station, TX 77843, USA; lydiachanmikang@tamu.edu (L.C.K.); mishajshah@gmail.com (M.S.); zina.trost@tamu.edu (Z.T.); 2 Department of Communication, University at Buffalo, The State University of New York, Buffalo, NY 14260, USA; araney@buffalo.edu; 3 Department of Communication, Cornell University, Ithaca, NY 14853, USA; asw248@cornell.edu; 4 Older People Rehabilitation and Cerebrovascular Medicine Research Laboratory, IRCCS Istituto Auxologico Italiano, 20149 Milan, Italy; v.mancuso95@gmail.com; 5 Department of Rehabilitation and Movement Sciences, Rutgers University, Newark, NJ 07102, USA; mbv42@shp.rutgers.edu; 6 Immersive Experience Laboratories, Birmingham, AL 35203, USA; corey.shum@goixl.com

**Keywords:** awe, virtual reality, chronic pain, mixed methods

## Abstract

As emerging media technologies, virtual reality (VR) applications are used to elicit emotional engagement across various contexts—entertainment, education, therapy, training, marketing, and fitness, to name a few—often with the hope of effecting behavioral change. Within the healthcare domain, VR technologies have been successfully leveraged to manage acute pain, but understanding of their efficacy in managing chronic pain remains limited. Current models emphasize the necessity of examining discrete effects of VR across multiple dimensions of pain, particularly its emotional impact. An emotion receiving increased attention by media scholars over the past few years is awe, the complex response to perceived vastness. Awe is a highly relevant, yet largely unexplored, emotion in chronic pain research, as it involves cognitive processes—vastness appraisals, feelings of connectedness, accommodation of new ideas—which may influence psychological processes shaping pain perception. To date, no study has examined the feasibility of inducing awe through VR among individuals with chronic pain. Accordingly, this preliminary study deployed a descriptive mixed-methods approach to examine the feasibility of utilizing a head-mounted display for VR-induced awe. Participants (*n* = 10) with CLBP viewed 360-degree virtual nature scenes depicting a mountain ascension, combined with music previously validated to elicit awe. Participants completed self-report measures before and after the VR session documenting pain intensity and emotional experiences. Semi-structured qualitative interviews were conducted following the post-session surveys. Results from both methods support the feasibility of inducing awe among individuals with chronic pain via virtual immersion, showing overall positive affective changes and reduction in frequency of self- and pain-related thoughts.

## 1. Introduction

Immersive experiences are a core, defining feature of most emerging media technologies. Arguably the most popular manifestation of these technologies is virtual reality (VR) (Kalyanaraman & Bailenson, 2019), which utilizes various technological capabilities to generate real-world or fantastical digital experiences (Gandhi & Patel, 2018). Fully immersive VR is delivered using head-mounted displays (HMDs), capable of crafting 360-degree virtual environments with digital imagery that can move in response to the user’s orientation and perspective, often enabling interaction with the digital environment (Trost et al., 2015). Given their relevance to gaming, mass-marketed consumer HMDs have been made extremely versatile through the combination of advanced display technologies, motion capture features, and integrated audio and sound design components (Saldana et al., 2020; Trost et al., 2015).

For the past few decades, media-effects scholars have documented how the immersion, presence, interactivity, and embodiment facilitated in and by VR experiences significantly impact emotions, cognitions, and behaviors within a variety of contexts, including entertainment, education, training, marketing, and fitness (for recent related summaries, see Lin et al., 2025; Martínez-Cano et al., 2023). Within healthcare contexts, VR interventions have repeatedly been shown to be effective tools for therapy (e.g., exposure, addiction, vision), motor skill recovery, cognitive assessment, and, increasingly, for acute pain management (Iqbal et al., 2024).

Pain is the most common reason people seek healthcare. Although undoubtedly subjective and highly variable, pain is widely defined as an “unpleasant sensory and emotional experience,” often in the context of “actual or potential tissue damage” (Raja et al., 2020). Pain is a biopsychosocial experience, with numerous factors shaping its perceptions, chronicity, and disability. VR interventions have consistently been demonstrated to be effective in addressing acute pain among pediatric and adult populations (Dreesmann et al., 2022; Gopalan et al., 2025; Lambert et al., 2020; Smith et al., 2020), in particular following medical procedures (e.g., burn debridement, wound management; Lou et al., 2025; Moreno-Martínez et al., 2025). Mechanistically, pain relief in these contexts is understood to be facilitated by the attentional demands of VR environments. That is, because the experience of pain demands attention (Eccleston, 1995), the distraction capabilities of VR experiences—especially immersive, interactive environments—enable situational pain relief (Dreesmann et al., 2022; Lambert et al., 2020). In contrast to the large body of evidence for acute pain, less research supports its application to chronic pain conditions, with studies largely limited to cognitive-behavioral education and movement promotion (Goudman et al., 2022; Trost et al., 2021; Wender & Trost, 2025). Critical commentaries on the “stagnant” nature of VR chronic pain research have highlighted an overreliance on distraction as the primary analgesic mechanism to the exclusion of other potentially meaningful dimensions of pain experience (Baker et al., 2022; Wender & Trost, 2025).

An alternative approach would be to address one or more of the psychological factors that exacerbate the sensory condition of pain. Notably, although emotion is integral to the definition of pain (Lumley et al., 2011), its role in VR pain research has received limited attention and has largely been confined to anxiety and distress (Baker et al., 2022). A focus on how emotions are generated and function within VR may help clarify and leverage a novel, understudied mechanism for advancing chronic pain VR research. Given its phenomenology, the emotion of awe would seemingly be a viable candidate for such research.

Awe is a complex emotion, defined as a psychological response to a stimulus perceived as vast, wondrous, or challenging, and which can evoke experiences of joy, wonder, fear, or even reverence (for overviews, see Allen, 2018; Chirico & Yaden, 2018). Awe often compels a need for cognitive accommodation while diminishing an individual’s sense of self, in turn eliciting mostly positive—though, at times, negative—affective reactions and feelings of connectedness (Keltner & Haidt, 2003). Reductions in self-salience and heightened feelings of connection are indicative of self-transcendent experiences (Yaden et al., 2017), during which attention to the self and one’s goals decreases and awareness of others and matters beyond the self increases. Given this, awe is often characterized as a self-transcendent emotion (e.g., Stellar et al., 2017).

Awe experiences vary across individuals (e.g., differences in trait openness to experience) and contexts (e.g., awe in response to vast physical beauty [e.g., aurora borealis] or vast physical destruction [e.g., earthquake or tsunami aftermath]). As a result, awe can be a conceptually ambiguous construct. Still, awe is typically described as an emotional experience that falls within the range of both pleasure and fear, often also tied to surprise (Chirico et al., 2016; Keltner & Haidt, 2003). In recent years, considerable interest has been directed to uncovering the transformative potential of awe to induce positive reappraisals of life (Piff et al., 2015; Prade & Saroglou, 2016), reduce aggressive tendencies (Yang et al., 2016), and increase prosocial behaviors (Piff et al., 2015; Prade & Saroglou, 2016).

Scholars have identified a variety of media depictions that are relevant to the elicitation of awe. Many prior studies (e.g., Bai et al., 2017; J. L. Gordon et al., 2017; Valdesolo & Graham, 2014) relied on video clips of inanimate nature scenery, such as “panoramic views of beautiful landscapes with waterfalls, deserts, oceans, large rivers, and high mountains” (Van Cappellen & Saroglou, 2012) to evoke awe in participants. Pictures, movies, and video games featuring space and space travel (A. M. Gordon et al., 2017; Possler et al., 2018; Silvia et al., 2015) have also been shown to trigger awe responses, as have portrayals of human-created objects (e.g., descriptions of overlooking Paris from the Eiffel Tower, Rudd et al., 2012; pictures of high buildings, Joye & Dewitte, 2016; certain music, Silvia et al., 2015; depictions across different points in a pregnancy, Saroglou et al., 2008; Van Cappellen & Saroglou, 2012). Experimental elicitations of awe through VR have remained largely unexplored until recent years, with Chirico and colleagues demonstrating that immersive virtual experiences have potential as awe-inspiring media forms (Chirico et al., 2016, 2018), and with Possler and colleagues demonstrating the same for certain digital games (Possler et al., 2018).

In the context of chronic pain, awe may offer a means of temporarily moving individuals out of a self-oriented stance. Critically, self-orientation is not intended as a moral or evaluative judgment but rather as an objective characterization of attentional and motivational focus. In the context of pain, this stance is evolutionarily prescribed; the function of pain is to command personal attention and inspire fear and self-protective behavior toward a vulnerable body (Eccleston & Crombez, 1999). Whereas this is critical in the case of acute pain, a prolonged and excessively fearful stance—supported either by explicit cognitive appraisal or hypervigilant nervous system networks (Ashar et al., 2022)—is thought to underpin chronic pain conditions. Specifically, this stance is central to the psychological constructs that have been most strongly implicated in the development and maintenance of chronic pain, disability, and pain-related social isolation, for example, pain catastrophizing (“It’s terrible and I think it’s never going to get any better”; e.g., Sullivan et al., 1995), pain-related fear (e.g., “I’m afraid that I might injure myself if I exercise”; e.g., Vlaeyen & Linton, 2000), and pain-related injustice perception (“No one should have to live this way”; e.g., Sullivan, 2008), each of which reflects a fearful, self-protective, and self-focused appraisal of pain and its potential impact. Accordingly, effective interventions in chronic pain explicitly aim to overcome this fearful, protective state (Ashar et al., 2022). Notably, established interventions such as Acceptance and Commitment Therapy (ACT) work in part by cultivating self-transcendence: cognitive defusion and “self-as-context” loosen identification with one’s own thoughts and feelings, whereas values-based action reorients the person outward (Hayes et al., 2006; McCracken & Vowles, 2014). Awe may engage these same self-transcendent processes through a single, immersive, embodied experience, while also disrupting the negative emotions (e.g., distress, fear, anger, sadness) commonly associated with chronic pain. With this possibility as a catalyst, we developed the current project as a first step for exploring VR-induced awe in chronic pain.

Chronic low back pain (CLBP) is a leading cause of disability worldwide (Wu et al., 2020), with acute back pain often transitioning into CLBP due to its persistent and recurrent nature (Gatchel et al., 2018). As argued above, awe may hold potential to shift an individual’s focus away from the self toward a greater connected whole while also reducing distress (Keltner & Haidt, 2003; Piff et al., 2015; Prade & Saroglou, 2016; Yang et al., 2016), possibly disrupting processes contributing to pain disability, such as pain-related fear and catastrophizing (Gatchel et al., 2007, 2018). This preliminary study is the first attempt, to our knowledge, to explore the feasibility of VR-induced awe among community individuals with CLBP. Further, the primary objective of this descriptive mixed-methods study is to assess the feasibility of a VR mountain journey as a means of inducing awe.

## 2. Materials and Methods

### 2.1. Study Design

This study used a convergent mixed methods design to integrate the quantitative findings with participants’ self-expressed feelings, thoughts, and opinions about their VR experience (Fetters, 2020). Given the novel nature of this endeavor, this design was selected to optimize a deeper understanding of participants’ VR experience (Fetters, 2020) in service of refinement of this research area. As detailed below, participants completed survey measures before and after a 3 min VR experience, along with a follow-up semi-structured, qualitative interview. All procedures described were approved by the Institutional Review Board at Texas A&M University.

### 2.2. Participants and Recruitment

Participants (*n* = 10) were recruited through online social media platforms and flyers and screened for eligibility. Screening was conducted over the phone by a member of the study team. Recruitment was conducted from January 2025 to May 2025. In total, 27 individuals were screened, with 10 individuals being eligible and selected for participation. All 10 selected participants completed all study related measures. Individuals were eligible to participate if they (1) self-reported having low back pain for longer than three months, (2) indicated having moderate to high pain intensity (at least 4/10; Melzack, 1987), (3) experienced pain interference in work or social activities (“Has your pain interfered in your work, school or social activities (i.e., prevents you from doing things you want to do)?” (Must be YES); PROMIS; Amtmann et al., 2010), and (4) had normal or corrected-to-normal visual function. Participants were excluded if they reported having any health conditions that impaired movement (e.g., arthritis, fibromyalgia, other major musculoskeletal limitations) that would make it difficult for them to stand during the VR experience. A cash payment of $45 was given upon completion of the session.

### 2.3. Procedures

Participants attended the research session in person at an on-campus facility. A trained research assistant guided participants through the informed consent procedure and then through the pre-VR session measures (see below). Immediately prior to and following the VR session, participants were asked to complete brief measures of current pain and state affect. Participants stood while viewing the VR stimuli. Following the VR experience, participants completed a series of assessment measures. Following this, the research assistant conducted and recorded a semi-structured qualitative interview assessing participant impressions of the VR experience. Recorded interviews were transcribed after the session, and audio recordings were deleted after transcription. Study sessions lasted between 60 and 90 min. No adverse events were reported, and no protocol deviations occurred across the 10 participant sessions. Data were missing for one incomplete survey item, as noted below.

### 2.4. VR Application and Stimulus

The VIVE XR Elite headset, an immersive HMD, was used to present a VR “mountain journey” (Trost et al., 2026), which lasted three minutes in total and contained several 360-degree scenes containing nature imagery previously validated to elicit awe among pain-free individuals (see Appendix A; Chirico et al., 2016, 2018). The VR experience opened with a scene depicting grassy, rolling hills, and transitioned to seven forested environments suggestive of a gradual ascent along a path up the mountain. At the top of the mountain, participants were “positioned” in a high-elevation setting featuring a mountaintop and sunset view. The experience concluded with two aerial images showing a similar, snowy mountain peak. The included imagery was developed based on previously validated images (Mancuso et al., 2024) and stimuli provided by Chirico and colleagues (Chirico et al., 2018). During the initial portion of the experience, ambient music (“Elysium”) from the soundtrack of the film “Gladiator” played, before transitioning to music previously validated to elicit awe (“Hoppípolla” by Sigur Rós; Silvia et al., 2015) during the final mountain scenes.

### 2.5. Pre-VR Exposure Measures

#### 2.5.1. Demographics, Back Pain History, VR Experience

Participants responded to basic demographic and back pain history items, per recommended guidelines in low back pain samples (Deyo et al., 2014). Participants were also asked about their previous experience using VR technology and to describe the details of prior VR use.

#### 2.5.2. McGill Pain Questionnaire, Short Form: Pain Rating Index (SF-MPQ PRI)

The Pain Rating Index of the short-form McGill Pain Questionnaire was used to assess average pain intensity over the two weeks prior to participating in the study (Burckhardt & Jones, 2003; Melzack, 1987). The PRI represented the sum of self-reported ratings across 15 adjectives that described sensory and affective dimensions of pain. Ratings were made on a 4-point scale from 0 (none) to 3 (severe), and scores could range from 0 to 45, where higher scores indicating greater average pain intensity (Hawker et al., 2011).

#### 2.5.3. Patient Health Questionnaire–9 (PHQ-9)

To further characterize the sample, depressive symptomatology was measured using the PHQ-9 (Kroenke et al., 2001). The PHQ-9 asks participants to rate the frequency with which they experienced each of nine listed symptoms within the past two weeks. Frequency scores ranged from 0 (not at all) to 3 (nearly every day). Total scores ranged from 0 to 27, with higher scores indicating greater depressive symptomatology.

#### 2.5.4. PROMIS Pain Interference–Short Form 6b (PROMIS-Interference)

Participants were given the PROMIS-Interference, which consisted of six items that asked them to rate the extent of pain-related interference with various relevant aspects of daily functioning on a 5-point scale from 1 (not at all) to 5 (very much) (Amtmann et al., 2010). Total scores ranged from 6 to 30, and greater scores indicated greater pain interference in the ability to function in daily life.

#### 2.5.5. Positive and Negative Affect Schedule (PANAS)

The PANAS was used to evaluate positive and negative state affect (Watson et al., 1988). Participants rated the extent to which they were currently experiencing the 20 affect terms on a Likert rating scale, ranging from 1 (very slightly or not at all) to 5 (extremely). The 20 terms were divided into two 10-item subscales, which were independently summed to determine separate scores for positive and negative affect.

#### 2.5.6. Pain Visual Analogue Scale (VAS)

Participants responded to the statement “Make a mark along the line that corresponds to how much pain you are currently feeling” on a 10 cm line, with each end of the line representing “No Pain” and “Worst Pain Imaginable,” respectively (Scott & Huskisson, 1976). Two members of the research team independently measured the distance between the beginning of the line (in cm) and the written mark, and then cross-compared measurements in order to finalize scores of current pain intensity, where higher scores indicated greater pain.

### 2.6. Post-VR Exposure Measures

Immediately following the VR experiences, participants once again completed the PANAS and VAS scales, as well as each of the following.

#### 2.6.1. Differential Emotions Scale (mDES)

A modified version of the Differential Emotions Scale (mDES) was administered to examine emotional responses to the VR experience (Shiota et al., 2006; Yaden et al., 2019). Participants were asked to reflect on how they felt while experiencing the VR stimuli using a list of 11 emotions rated on a 7-point scale ranging from 1 (not at all) to 7 (extremely). For creating the 11 item mDES, nine items were selected from the original 30-item list validated by Yaden et al. (2019), with the addition of two items: amazement and nostalgia. Amazement was included as a near-synonym of awe to capture the target emotion more sensitively, whereas nostalgia was included because immersive virtual nature experiences, and the autobiographical memories they evoke, commonly elicit nostalgia (Mancuso et al., 2023; Schöne et al., 2019), a self-relevant emotion linked to social connectedness and meaning (Routledge et al., 2011). Including it allowed us to characterize the broader affective response and to differentiate awe from reminiscence-driven positive affect.

#### 2.6.2. Awe Experience Scale (AWE-S)

The AWE-S consisted of 30 items, divided into six subscales of five items each, corresponding to the six facets of the awe experience specified by Yaden et al. (2019): vastness, accommodation, self-loss, time perception, physiological changes, and connectedness (Yaden et al., 2019). Each item was rated on a 7-point scale of 1 (strongly disagree) to 7 (strongly agree).

#### 2.6.3. Treatment Evaluation Inventory (TEI)

The Treatment Evaluation Inventory (TEI) evaluated the acceptability and perceived feasibility of an intervention and consisted of nine items assessing positive and negative attitudes toward a treatment (Kelley et al., 1989). In the current study the TEI was used to evaluate the acceptability of using the VR stimulus as part of pain intervention (Trost et al., 2022, 2026). Participants rated their agreement with each statement on a 5-point scale of 1 (strongly disagree) to 5 (strongly agree), with ratings summed to calculate scores ranging from 9 to 45. Higher scores reflected higher treatment acceptability.

#### 2.6.4. Simulation Sickness Questionnaire

The Simulation Sickness Questionnaire was used to assess motion-sickness-related symptoms that occurred during the VR experience (Kennedy et al., 1993). Participants used a 4-point scale ranging from 0 (none) to 3 (severe) to rate the extent to which they experienced a list of 16 different symptoms. Ratings are summed to provide a total score ranging from 0 to 48, with higher scores indicating greater severity of motion sickness symptoms as a result of the VR experience.

#### 2.6.5. Semi-Structured Qualitative Interview

A semi-structured qualitative interview was conducted following the VR experience to explore the feelings, thoughts, and opinions participants had during or with respect to their VR experience. Each interview was conducted using an interview guide (see Appendix B), by the first, second, and third authors. Interviewers were trained on qualitative interviewing methods by two senior members of the research team (Goodell et al., 2016) and instructed to ask probing questions to capture in-depth responses from each participant. The interview guide was developed by a senior author and pilot tested among members of the research team. Revisions were made after pilot testing, resulting in a 13-question guide. Interviews ranged from 10 to 40 min in length.

### 2.7. Data Analysis

#### 2.7.1. Quantitative Analysis

Given the small sample size, descriptive statistics were generated for quantitative measures. For AWE-S items, the proportion of participants endorsing agreement (a rating of 5 or higher on a 7-point scale, where 4 represents neutral) was calculated for each item. Participants were classified as agreeing with a subscale if they endorsed at least three of its five items. Responses to each mDES item were analyzed individually, with higher scores indicating a stronger endorsement. To facilitate meaningful interpretation alongside qualitative measures, mDES items were also categorized as “moderate” for rankings between 2 to 4 and “strong” for rankings between 5 to 7. Because the 11-item mDES used in this study was adapted from the validated 30-item scale, results are reported only as exploratory, item-level descriptions. Changes in the VAS were calculated in centimeters and shown as positive or negative numbers representing directional change following the VR experience. PROMIS-Interference total scores were converted to *T* scores and interpreted using official scoring tables and severity thresholds (Cella et al., 2019; HealthMeasures, 2023).

#### 2.7.2. Qualitative Analysis

The study was grounded in basic qualitative research, which is characterized by constructivism and aims to understand how people make sense of their lived experiences (Merriam & Tisdell, 2015). Thematic analysis is well-suited for basic qualitative research and provided a systematic, flexible, and inductive analytic approach, as well as depth to our understanding of the participants’ VR experiences (Braun & Clarke, 2006, 2023). The analysis team consisted of two student research assistants with VR research experience, a physical therapist and early career pain researcher with substantial qualitative research training but without prior VR research experience, and an experienced VR and pain researcher. As outlined by Braun and Clarke (2006), the thematic analysis started by becoming familiar with the data by reading the full transcripts. Next, initial codes were generated by the two student coders who highlighted and conceptually labeled segments of text. This occurred independently using separate Word documents and Excel files to reflect on personal experiences, reactions, and preconceptions, and collaboratively to share reflections and appreciate new and shared interpretations of data. The student coders collaboratively grouped similar codes and iteratively updated initial codes by referring to the original transcripts. Throughout this process, student coders deliberately aimed to identify excerpts from the text or examples that were counter to the proposed themes. Negative or divergent cases were included to illustrate a range of perspectives from the current study (Crabtree & Miller, 2023). Initial themes were generated and reviewed to ensure the themes worked in reference to original transcripts, codes, and the dataset. Initial themes were refined and collaborative links were identified through collaborative discussions across the full coding team (two student coders, a physical therapist and early career pain researcher, and an experienced VR and pain researcher). After initial themes were refined, conceptual links were identified by the full coding team through collaborative discussions. This iterative process led to defining and naming the themes and collaboratively producing the current report. This team-based approach was used to promote reflexivity through the analytic process (Crabtree & Miller, 2023). Throughout the analysis process, the student coders frequently met and engaged in reflexivity by journaling and meeting for discussion after coding for each transcript (Crabtree & Miller, 2023). These discussions included how researcher positions may influence data analysis (e.g., working in a VR research lab) and informed the decision to include team members who had less experience with this VR paradigm (a physical therapist and early career pain researcher). Due to the novel VR technology and the analysis being completed within members of a VR research laboratory, potential social-desirability biases exist.

#### 2.7.3. Data Integration and Synthesis

Quantitative and qualitative data were collected and analyzed in parallel (Fetters, 2020; Guetterman et al., 2015, 2021). Data were integrated by comparing related quantitative measures to each theme found in the qualitative data. A joint display (see below) summarizing awe experiences was created by pairing a representative quote from each participant with their quantitative score on the VAS, endorsed mDES items, and endorsed AWE-S subscales (Guetterman et al., 2021). This was accomplished by team members visualizing the qualitative and quantitative data side by side on a table, and pairing data together accordingly.

#### 2.7.4. Assessing Feasibility

The primary interest of this study was to examine the feasibility of using VR for awe-induction. This exploratory approach was well-suited to a novel concept, VR-induced awe in individuals with CLBP, that has not yet been explored (Bowen et al., 2009). The feasibility of using VR to induce awe was assessed through outcomes on the AWE-S, mDES, and TEI.

Since prior awe literature has not established metrics for the AWE-S and mDES, feasibility in this study was defined as the presence of self-reported awe in the majority of the sample. As mentioned, awe can also be described as a self-transcendent emotion. This study did not directly measure self-transcendence; however, accommodation, self-loss, connectedness, and vastness subscales evaluated components of transcendence defined in the literature (Yaden et al., 2017). In line with this literature, these subscales were also used to examine the presence of possible self-transcendent experiences. The TEI was used alongside the AWE-S and mDES, for assessing the overall feasibility of the VR experience. Majority agreement with positive TEI items (“I would like this VR procedure if it were used as part of treatment.”) and absence of agreement with negative items (“I believe this VR procedure is likely to harm or injure my body.”) indicated feasibility in this study.

## 3. Results

### 3.1. Participant Demographics

All ten participants in the study sample identified as White and non-Hispanic; six participants self-identified as female and four as male, with ages ranging from 22 years to 84 years. Self-reported pain duration was at least one year, with a range of 1–30 years. Annual income across participants was varied, ranging from $20,000–$29,999 to over $100,000 per year. All participants reported completion of at least some college. PHQ-9 scores ranged from 2 to 19, indicating minimal to moderate/severe symptoms. PROMIS-Interference scores ranged from 11 to 29, with *T* scores indicating mild (53.8) to severe (74.4) levels of pain interference (see above Section 2.7.1 for scoring procedures). Finally, pain intensity (SF-MPQ PRI; Melzack, 1987) scores ranged from 1 to 27 over the past two weeks. Although some participants reported low SF-MPQ PRI scores, all satisfied the inclusion criterion of an average pain intensity above 4 during the two weeks prior to screening. Of the ten participants, seven reported previous exposure to VR technology, with five reporting a current VR usage frequency of less than 1 to 2 times a year (Table 1).

### 3.2. Theme 1: Awe Experiences Contributing to a Shared Sense of Perceived Vastness, Connectedness, and Wonder

On the AWE-S, eight out of ten participants endorsed agreement with at least two subscales (Table 2), with nine participants endorsing agreement with the subscale representing feelings of perceived vastness. Similarly, participants communicated awe appraisals during the qualitative interview (Table 3) through reported feelings of smallness, perceived vastness, and connections to a greater whole. Participants endorsing agreement with multiple AWE-S subscales also reported similar feelings in their qualitative responses, as shown in Table 3. Awe-related mDES items (i.e., awe, wonder, amazement) were endorsed by a larger majority of the participants than non-awe-related items (i.e., nostalgia, hope, joy, pride).

### 3.3. Theme 2: Positive Emotional Engagement, Enjoyment, and Varying Interpretations of the VR Environment

#### 3.3.1. Overall Positive Reactions to the VR Stimulus

**Figure 1:**
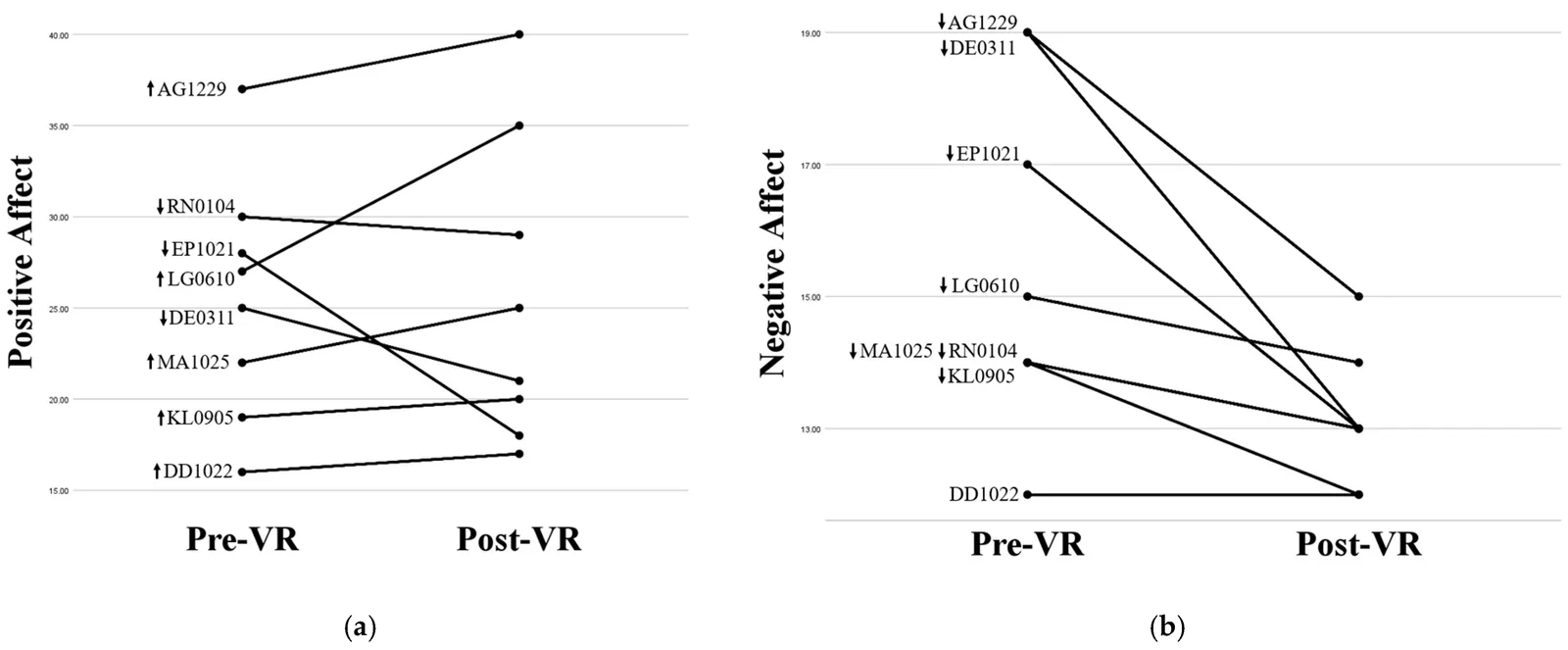
(**a**) Positive and (**b**) Negative Affect Scores Before and After the VR Experience. Up arrows represent an increase in positive or negative affect score, and down arrows represent a decrease. Participants DT0604 and RE0704 have been removed due to inattentive responding.

Participant reactions to the VR environment varied but were overall positive in nature. General enjoyment and fondness were shared across participants toward particular elements of the stimulus, such as the mountain imagery, music, and snow falling from above. Enjoyment of the stimulus was shared along with feelings of joy, happiness, and relaxation during the VR experience. Qualitative statements were consistent with scores on the PANAS, where we observed an elevation in positive affect scores and a decline in negative affect scores following VR exposure in the majority of the participants (Figure 1a,b).

Quantitative responses on the PANAS were visually scanned for inattentive responding, which revealed two outliers on the PANAS pre- and post- questionnaires. Inattentive responding was defined as inappropriately similar or like responses across survey items. Participant ID’s DT0604 and RE0704 appeared to respond with the same values across pre- and post- PANAS items (DT0604: Pre-PANAS Positive Affect Score = 34, Post-PANAS Positive Affect Score = 43, Pre-PANAS Negative Affect Score = 26, Post-PANAS Negative Affect Score = 38; RE0704: Pre-PANAS Positive Affect Score = 34, Post-PANAS Positive Affect Score = 10, Pre-PANAS Negative Affect Score = 14, Post-PANAS Negative Affect Score = 11). For this reason, scores from DT0604 and RE0704 were omitted from Figure 1. For positive affect (Figure 1a), scores (Pre-VR (*n* = 8): range: 16 to 37, *M* = 26, IQR = 9.75; Post-VR (*n* = 8): range: 17 to 40, *M* = 23, IQR = 15) increased in five and decreased in three of eight participants (range: −35.71 to 29.63; IQR = 25.09; *M* = 5.76). For negative affect (Figure 1b), scores (Pre-VR (*n* = 8): range: 12 to 19, *M* = 14.5, IQR = 4.5; Post-VR (*n* = 8): range: 12 to 15, *M* = 13, IQR = 1.5) decreased in seven and remained the same in one of eight participants (range: −31.58 to 0.00; IQR = 16.12; *M* = −10.71).

*“I thought it was cool. I enjoyed it. I’m a really ‘big with nature’ person in general, so kind of being in what was in the VR space was really enjoyable for me.”*

(MA1025)

Similarly, participants reported strong feelings of amusement and moderate to strong feelings of joy on the mDES (Table 4), as well as a complete absence of endorsement (*n* = 10) for anger or disgust and nearly so (*n* = 9) for sadness.

#### 3.3.2. Interpretations of the Mountain Journey

Many participants shared their interpretations of the VR environment they experienced. Although participants were not informed prior to the experience that they would be viewing a simulated mountain journey, some noted their curiosity regarding the path they were being led on. Others noticed ski-lifts near the mountain peaks and had mixed opinions about the presence of other people on their mountain journey. A majority of participants found that the stimuli resembled past traveling experiences, media they were fond of, or key life milestones.

*“The birds flying over prompted a memory of my wedding because when we got married, two Mallards flew over us.”*

(DT0604)

Fittingly, participants reported moderate (*n* = 2) to strong (*n* = 5) feelings of nostalgia on the related mDES item (Table 4).

### 3.4. Theme 3: Somatic Experiences and Reductions in Pain-Related Thoughts

Participants expressed some level of immersive experience, either by indicating increased presence, noting the effects of the 360-degree environment, or through somatic experiences.

#### 3.4.1. Indications of Engagement, Captivation, and Presence

Participants commonly reported feeling present and immersed within the VR scenes, sometimes giving rise to a desire to prolong their stay in the session.

*“Oh, I thought [that I] didn’t want [the VR] to stop.”*

(DE0311)

Somatic experiences were reported by some, resulting in physical changes felt within their body. The ability to turn and view their surroundings in 360 degrees was also noted, possibly contributing to their immersive experiences.

*“I think there was some surprise when you got to go from being on the ground to being in the air, which was cool, like being at elevation. And so I know that those things do give you, you know, even a feeling of a lightness, which was neat. I like that.”*

(DD1022)

#### 3.4.2. Overall Reduction in Pain-Related Thoughts

The majority of participants observed that they had little to no thoughts about their pain during the VR session, indicating a shared reduction in pain-related thoughts across the sample.

*“I didn’t really think about [my pain].”*

(DD1022)

VAS scores (Table 3; Pre-VR (*n* = 10): range: 0.50 to 7.55, *M* = 2.23, IQR = 1.63; Post-VR (*n* = 10): range: 0.35 to 6.45, *M* = 1.53, IQR = 2.01) declined following the VR experience in seven of the 10 participants and increased in three of 10 participants (range: −2.10 to 0.45; IQR = 1.66; *M* = −0.95). Participants who reported some increase in pain on the VAS also discussed negative feelings towards standing or having overall low pain severity on the day of the session.

*“And especially like when you stand up… it’s… there. It’s not horrible, but, you know, it’s there. You can feel it.”*

(AG1229)

*“I’m having very little pain. I mean. I always have a slight discomfort, but never, but it’s not painful today, really.”*

(EP1021)

*“It feels better to move around versus just standing.”*

(KL0905)

### 3.5. Theme 4: Attitudes Towards VR Interventions for Chronic Pain

#### 3.5.1. Positivity and Enthusiasm for Pain-Related VR Interventions

In interview responses, the overall attitude from participants was positive about the future application of VR as an intervention for chronic pain, with some sharing their hopefulness for the future. On the TEI (Table 5), nine out of 10 participants endorsed having a positive reaction to the VR experience, and all participants would be “willing to use this procedure if prescribed or recommended by a physician.”

*“I think this is another avenue of being able to give comfort to those individuals who could just, you know, enjoy being where the virtual reality scenes take you.”*

(DE0311)

#### 3.5.2. Criticisms and Areas for Improvement

Although largely positive, some notable criticisms were expressed regarding the quality of the VR stimulus and discomfort from the equipment used. Of those sharing uncertainty about the applications for pain-related VR interventions, some also had small increases in their VAS scores. Participants noted that they enjoyed the stimulus but were aware of being in a virtual environment. Scores on the TEI and simulation sickness questionnaire indicated a positive reaction to the VR environment in nearly every participant (Table 5), but revealed low levels of discomfort in five participants (Table 6 and Table 7).

*“I’m not really sure what you’re doing. I’m having trouble understanding how the VR helps pain. But then I don’t have any pain right now, so I can’t say what it does.”*

(EP1021)

Of the seven participants who reported previous VR exposure, one (RE0704) reported that their responses were strongly influenced by a personal preference for different, previously tried VR equipment. Outside of personal distaste for the equipment used to conduct the study, this participant notably reported high anticipation and endorsement of the potential future applications of VR as an intervention for chronic pain. This participant’s prior experience seemed to also influence their responses throughout the session.

*“[The other headset is] just a better experience, and you’d get more positive feedback from people with just a better headset. You’d get a better experience with the people that you’re working with.”*

(RE0704)

### 3.6. Feasibility

Our mixed-methods findings revealed strong feelings of awe in the majority of the sample, and overall feasibility of the VR mountain journey for awe induction. On the AWE-S, eight of 10 participants endorsed agreement with at least two subscales, and nine of 10 endorsed agreement with at least one subscale. In terms of transcendence, the vastness subscale was endorsed by nine of 10 participants, self-loss subscale was endorsed by five of 10, connectedness was endorsed by three of 10, and accommodation was endorsed by one of 10. On the mDES, five of 10 participants endorsed agreement with all awe-related items (i.e., “awe,” “wonder,” “amazement”) and nine of 10 participants endorsed agreement with at least one awe-related item. On the TEI (Table 5), nine out of 10 participants endorsed having a positive reaction to the VR experience, and all participants would be “willing to use this procedure if prescribed or recommended by a physician.” These quantitative findings were echoed in qualitative interviews, where participants shared reports congruent with feelings of awe, and positivity related towards their VR experience.

## 4. Discussion

As a fast-evolving media technology, VR has a unique potential to impact the emotions, cognitions, and behaviors of users via immersion, interactivity, and embodiment processes. Media-effects researchers have documented how these effects can be harnessed for a variety of user benefits, including in the healthcare sector (e.g., exposure therapy, Carl et al., 2019; motor skill recovery, Laver et al., 2017). Of particular relevance for the current study, VR interventions can help effectively manage acute pain (e.g., Dreesmann et al., 2022; Gopalan et al., 2025), but considerably less is known about their application to chronic pain conditions. For persons living with chronic pain conditions such as CLBP, the experience of physical pain is often shaped and compounded by psychological factors characterized by a self-oriented, protective stance toward pain. As a self-transcendent emotion, awe (i.e., the psychological response to perceived vastness; e.g., Chirico & Yaden, 2018) holds promise as a means for helping individuals with CLBP to overcome such fearful, protective states. Moreover, VR holds promise as a technology to powerfully elicit awe through immersive and embodied experiences. To our knowledge, this was the first study to explore VR induced awe in individuals with CLBP.

As part of this preliminary investigation, we tested the feasibility of using a 3 min virtual mountain journey designed to elicit awe in an initial sample of individuals (*n* = 10) with CLBP. The results of our mixed method study were highly encouraging. First, participants did indeed experience awe, as they nearly unanimously reported experiencing vastness (nine of 10 on the AWE-S scale) along with awe, wonder, or amazement (six, seven, and seven of 10, respectively, on the mDES). Further, participants who reported experiencing vastness also felt other aspects of awe, such as self-loss and connectedness. Similar themes emerged in qualitative reports, as participants described strong feelings of connectedness, immersion, and vastness from being in the 360-degree VR environment. Furthermore, awe was endorsed to a greater extent across the sample than other positive emotions (e.g., joy, hope, pride), suggesting that the potential of the VR mountain journey for awe-induction was greater than it was for general positive affect. Results on the TEI revealed unanimous support for future, similar pain interventions. These findings support the presence of awe within the sample and, therefore, the feasibility of using VR HMDs to induce awe.

In terms of general emotional responses, although elevation in positive affect following the VR exposure was mixed across the sample, overall, participants endorsed moderate levels of amusement, hope, and joy. Feelings of nostalgia and overall enjoyment were also common, as reflected in both the scale responses and follow-up participant interviews. Importantly, following the VR experience, we observed a consistent decline in negative affect and an absence of fear and disgust (per the mDES). This observed decrease in negative affect is congruent with common awe appraisals resulting from nature stimuli (Yaden et al., 2019).

Given the general positive experiences reported by the sample, it is perhaps unsurprising that attitudes towards the future potential of VR interventions for pain were also generally positive. Notably, participants expressing greater enjoyment of the session also reported more enthusiasm for pain-related VR interventions. Though some participants expressed uncertainty when discussing these interventions in the qualitative interview phase, their treatment evaluation (TEI) scores showed openness towards future participation in similar interventions or research studies. Prior VR experience with other, preferred HMDs appeared to influence at least one participant’s (RE0704) responses; however, this familiarity did not appear to diminish overall enjoyment or enthusiasm for future VR interventions.

As in previous research, exposure to the VR stimulus was followed by a decrease in participants’ current pain (Dreesmann et al., 2022; Eccleston, 1995; Lambert et al., 2020), which was endorsed by a majority of participants both quantitatively (i.e., seven of 10 reported decreased VAS scores) and in qualitative interviews (i.e., majority of participants reported that they had little to no thoughts about their pain). Although VR has previously been validated as an attentional tool for distraction in acute pain populations (Dreesmann et al., 2022; Eccleston, 1995; Lambert et al., 2020), no study to date has specifically examined the effects of VR-induced awe on individual’s pain perception, either in the context of acute or chronic pain. However, in addition to its apparent distracting value, we observed indicators of self-transcendent effects of the VR stimulus, with reports of self-diminishment, perceived vastness, and a higher sense of connectedness (i.e., awe). Moreover, participants with the greatest reduction in self-reported pain reported endorsements of awe. Although our findings do not provide causal evidence, we highlight a potential avenue for future work examining the relationship between awe and psychological factors associated with chronic pain. Specifically, future research may examine the potential of VR-induced awe to move individuals out of a maladaptively protective self-oriented state, which may reduce pain severity and increase uptake of other therapeutic interventions targeting pain-related disability. Importantly, this avenue remains strictly theoretical and is not directly supported by the present findings. Future studies should also focus on isolating potential transcendent effects from general positive affect and distraction.

Along with the encouraging results, we must acknowledge some limitations in the study. The sample size is admittedly too small (*n* = 10) and too homogeneous (all White and non-Hispanic, with some college and no mobility limitations) to draw generalizable conclusions, but the promising results can serve as an initial “proof of concept” for the feasibility of VR-induced awe in chronic pain. This study is fundamentally exploratory, and changes observed in VAS scores could be unrelated to participant VR experiences. Also, although vastness was universally perceived at a high level, other aspects of awe were less consistently reported. Finally, additional testing must be completed to more confidently state that the psychological mechanism driving the observed effects is the self-transcendent qualities of the awe experience rather than simply improved positive affect or attentional distraction.

## 5. Conclusions

Despite these limitations, we are encouraged by the results of this initial study. Across multiple measures, participant responses support the feasibility of inducing awe using VR stimuli in this population. Participants’ overall experience with the VR mountain journey was nearly unanimously positive, emphasizing enthusiasm and hopefulness for the availability of similar treatment options in the future. Furthermore, participants who reported the largest decreases in pain following the session also reported intense awe experiences. Overall, these results provide support to further explore the feasibility of utilizing HMDs for awe-induction, which may influence the experiences of individuals with chronic pain. These findings highlight a potential avenue for future work examining the relationship between awe and psychological outcomes associated with chronic pain. Although such studies may be difficult given the complex nature of awe as a quantifiable construct, we emphasize further investigation in this direction with hopes of opening new possibilities of incorporating awe as an element of future treatment and interventions for chronic pain conditions.

## Figures and Tables

**Table 1:** Participant Demographics, Pain History, and Prior VR Usage.

Participant ID	Gender	Age	Pain Duration	SF-MPQ PRI	PHQ-9	PROMIS-Interference	Prior VR Use
AG1229	Female	78	1	3	2	14	Y
DD1022	Male	43	14	4	2	14	Y
DE0311	Female	79	3	20	19	29	Y
DT0604	Female	25	16	27	15	29	N
EP1021	Male	84	5	1	9	15	N
KL0905	Female	39	10	4	3	11	Y
LG0610	Male	54	30	6	14	19	Y
MA1025	Female	22	5	10	9	14	Y
RE0704	Male	54	28	10	12	28	Y
RN0104	Female	68	10	16	8	13	N

Note. Age and pain durations are represented in years. SF-MPQ, PHQ-9, and PROMIS-Interference totals were calculated by summing scale items (15, 9, and 6 items, respectively). Prior VR use is indicated as Y (Yes) or N (No).

**Table 2:** AWE-S scores on each subscale and item.

Subscale	Item	Agreement with:	
		Item	Subscale
Time Perception	I had the sense that a moment lasted longer than usual.	5/10	2/10
	I felt my sense of time change.	3/10	
	I experienced the passage of time differently.	3/10	
	I sensed things momentarily slow down.	3/9	
	I noticed time slowing.	2/10	
Self-Loss	I felt that my sense of self diminished	4/10	5/10
	I felt my sense of self shrink	3/10	
	I felt a reduced sense of self	3/10	
	I felt my sense of self become somehow smaller	3/10	
	I felt small compared to everything else	2/10	
Connectedness	I felt a sense of communion with all living things	6/10	3/10
	I had the sense of being connected to everything	4/10	
	I experienced a sense of oneness with all things	4/10	
	I had a sense of complete connectedness	3/10	
	I felt closely connected to humanity	2/10	
Vastness	I felt that I was in the presence of something grand	9/10	9/10
	I experienced something greater than myself	8/10	
	I felt in the presence of greatness	8/10	
	I perceived something that was much larger than me	8/10	
	I perceived vastness	8/10	
Physiological Changes	I felt my eyes widen	4/10	2/10
	I felt my jaw drop	3/10	
	I gasped	1/10	
	I had chills	1/10	
	I had goosebumps	0/10	
Accommodation	I tried to understand the magnitude of what I was experiencing	7/10	1/10
	I felt challenged to mentally process what I was experiencing	5/10	
	I found it hard to comprehend the experience in full	2/10	
	I felt challenged to understand the experience	2/10	
	I struggled to take in all that I was experiencing at once	1/10	

Note. The AWE-S had a total of six subscales and thirty items, with 5 items in each subscale. Each item was ranked on a scale of 7-point scale of 1 (strongly disagree) to 7 (strongly agree). Participants were identified as endorsing agreement with an item if they ranked the item above a 4 (neutral). Majority agreement was documented with each subscale, if a participant endorsed agreement on at least three out of five subscale items. Proportions represent the total number of participants endorsing agreement on either an item or subscale. One item (“I sensed things momentarily slow down”) had missing data for participant LG0610, as it was skipped during completion of the survey measures.

**Table 3:** Comparing changes in pain scores following the VR experience to awe-related item endorsements, and qualitative findings for each participant.

Participant	Pre-VR	Post-VR	∆VAS	mDES	AWE-S	Related Interview Quotes
RN0104	3.90	1.80	−2.10	Awe Amazement Wonder	Time Self-Loss Connectedness Vastness Physiological	Well, kind of like in some of your questions, then you realize the vastness of it, you know, and then you look down, and then you realize, “Oh!” Very awe inspiring. It was good.
DE0311	2.05	0.35	−1.70	Awe Amazement Wonder	Self-Loss Vastness	I suppose I was thinking less about myself and more about the creator and the beauty of the things that [God] created.
MA1025	2.04	0.48	−1.56	Awe Amazement Wonder	Connectedness Vastness	So it’s, like, it was almost motivating.
DT0604	7.55	6.45	−1.10	Awe Amazement Wonder	Time Self-Loss Vastness	I think maybe… where there was more like rolling fields, open. That one felt vast and comfortable.
LG0610	2.85	1.75	−1.10	Awe Amazement Wonder	Self-Loss Connectedness Vastness	Seeing these scenes as the resolution increased, the intensity of the wellness, I guess, the feelings of wellness are content, or just happy and joyful and reverent, since reverence to me is religious attachment. So, if you’re a God-fearing person, then you think God created the world, and that is a very beautiful thing.
DD1022	1.65	0.85	−0.80	-	Self-Loss Vastness	I think it’s… there’s rising scales and there’s all kinds of things in there that I think were a good choice, or whether they’re intentional or not, I think it was fitting and I think it, yeah, it draws out that sense of awe and the idea of not questioning… I don’t know, it puts at ease a questioning and wondering mind.
RE0704	2.40	2.35	−0.05	-	-	-
AG1229	0.50	0.55	0.05	Amazement Wonder	Vastness	-
EP1021	1.00	1.10	0.10	Amazement Wonder	Vastness Accommodation	It was very interesting. It was amazing that I could look all the way around me, basically, and see everything.
KL0905	2.65	3.10	0.45	Awe	Vastness Physiological	-

Note. Each row represents responses from one participant. Negative ∆VAS scores represent a decrease and positive values represent an increase in pain following the VR experience. AWE-S subscales for which participants rated majority agreement are listed. Awe-related mDES items (i.e., awe, wonder, amazement) are listed if participants rated the item higher than the scale midpoint (i.e., 4). Representative quotes for each participant were chosen and displayed next to their quantitative results.

**Table 4:** Number of participants endorsing agreement with mDES items.

Item	Not at All (1)	Moderate (2–4)	Strong (5–7)
Anger	10/10	-	-
Disgust	10/10	-	-
Pride	1/10	9/10	-
Sadness	9/10	1/10	-
Amusement	2/10	1/10	7/10
Nostalgia	3/10	2/10	5/10
Awe	1/10	3/10	5/10
Hope	2/10	6/10	1/10
Joy	1/10	6/10	3/10
Amazement	1/10	2/10	7/10
Wonder	1/10	3/10	6/10

Note. Items are ranked on a scale of 1–7, with 1 representing zero extent to which the item is felt. Items ranked between 2 to 4 were categorized as “moderate” and items ranked between 5 to 7 were categorized as “strong.” Proportions represent the number of participant scores ranked as not at all, moderate, or strong.

**Table 5:** Participants endorsing agreement with TEI items.

Item	Participant Agreement
I would be willing to use this procedure if prescribed or recommended by a physician.	10/10
I would like this VR procedure if it were used as part of treatment.	9/10
Overall, I have a positive reaction to this VR experience.	9/10
I believe this VR procedure will help treatment be effective	6/10
I would find this VR experience to be an acceptable and appropriate part of a pain intervention.	6/10
I believe it would be acceptable to use this VR procedure with individuals who cannot choose treatments for themselves.	5/10
I believe that treatment including this VR procedure is likely to result in permanent improvement.	4/10
I believe that I will experience discomfort during this VR procedure.	3/10
I believe this VR procedure is likely to harm or injure my body.	0/10

**Table 6:** Participants endorsing agreement with Simulation Sickness Questionnaire items.

Item	Rating > 0
General discomfort	5/10
Fatigue	1/10
Headache	1/10
Eye strain	2/10
Difficulty focusing	4/10
Salivation increasing	0/10
Sweating	0/10
Nausea	0/10
Difficulty concentrating	3/10
Fullness of the head	0/10
Blurred vision	1/10
Dizziness with eyes open	0/10
Dizziness with eyes closed	0/10
Vertigo (experienced as loss of orientation with respect to vertical upright)	1/10
Stomach awareness (indicates a feeling of discomfort just short of nausea)	1/10
Burping	0/10

**Table 7:** Simulator Sickness total ratings for each participant.

Participant	Summed Total Score
AG1229	0
DD1022	4
DE0311	0
EP1021	2
KL0905	3
LG0610	2
MA1025	5
RN0104	0

*Note.* Items were ranked on a 4-point scale ranging from 0 (*none*) to 3 (*severe*) to rate the extent to which they experienced a list of 16 different symptoms (see Table 6). Ratings were summed to provide a total score ranging from 0 to 48.

**Figure A1:**
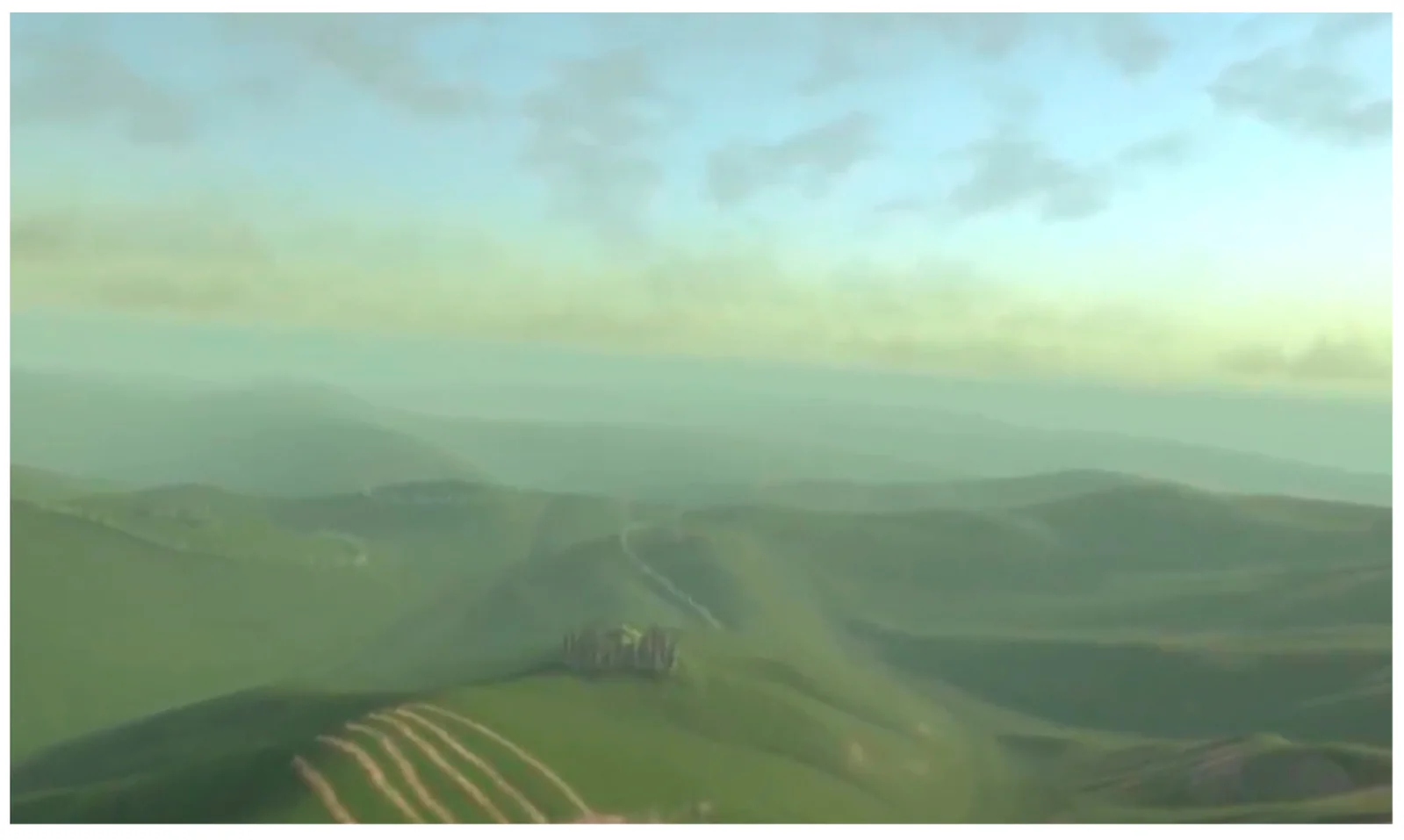
Scene 1.

**Figure A2:**
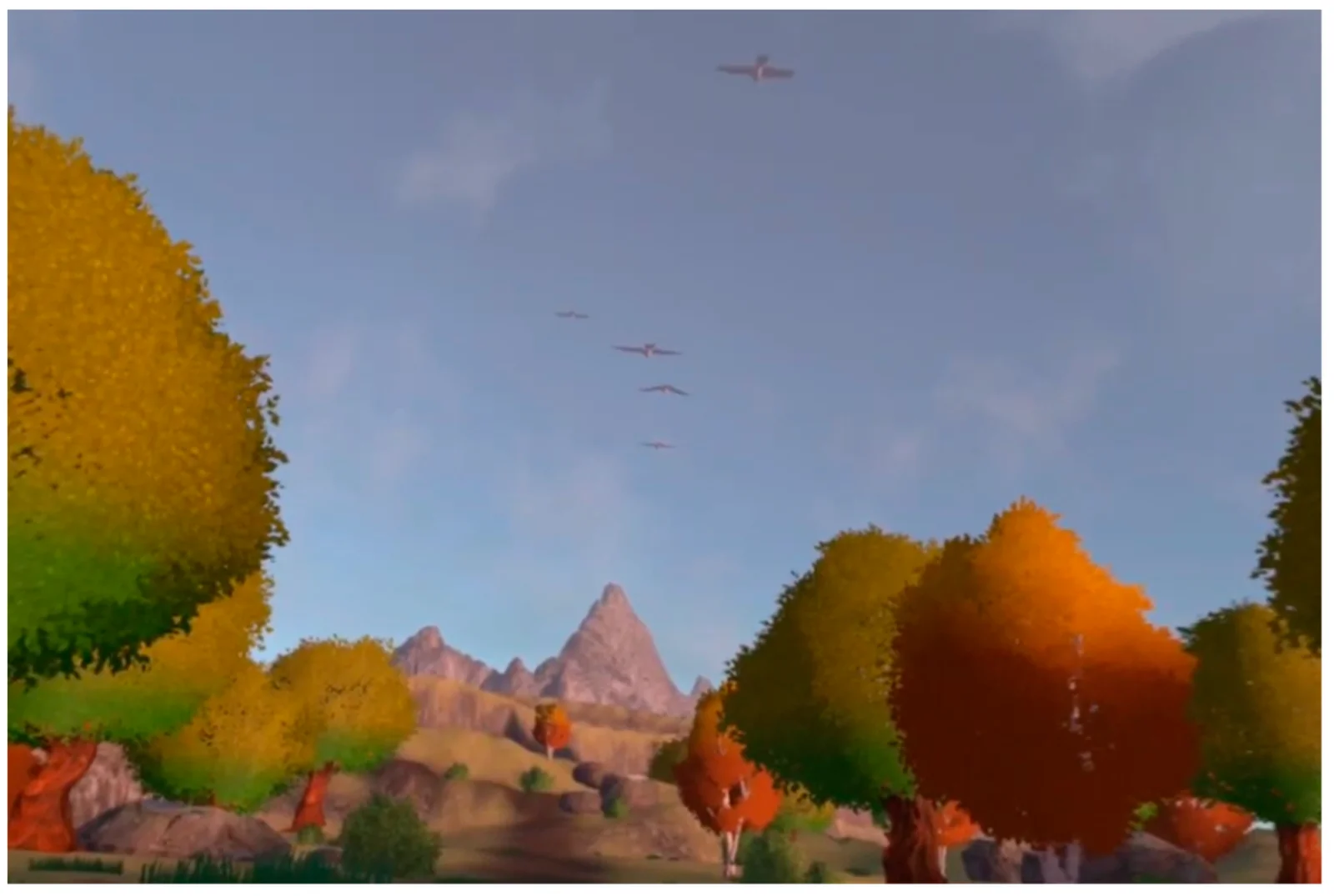
Scene 2.

**Figure A3:**
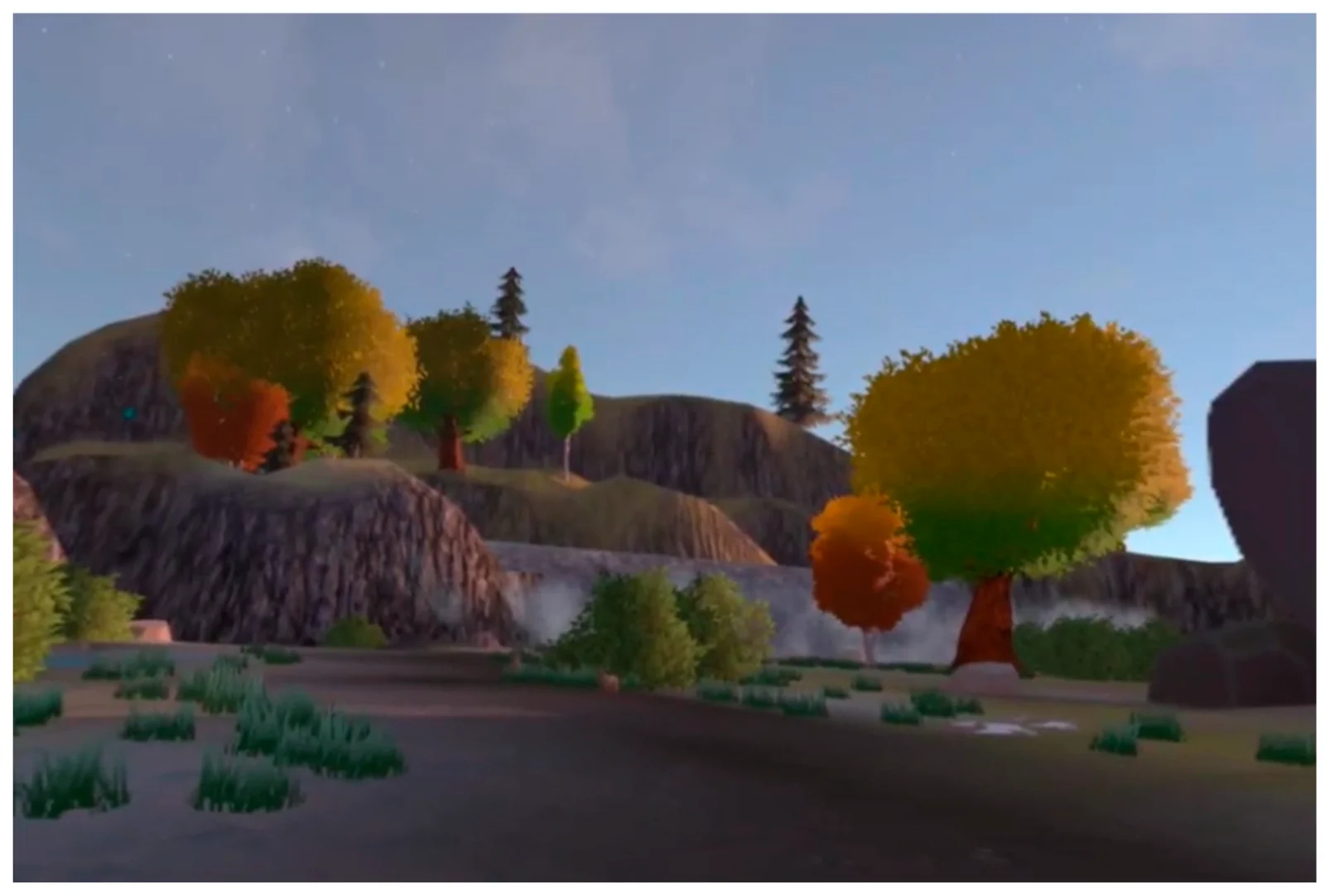
Scene 3.

**Figure A4:**
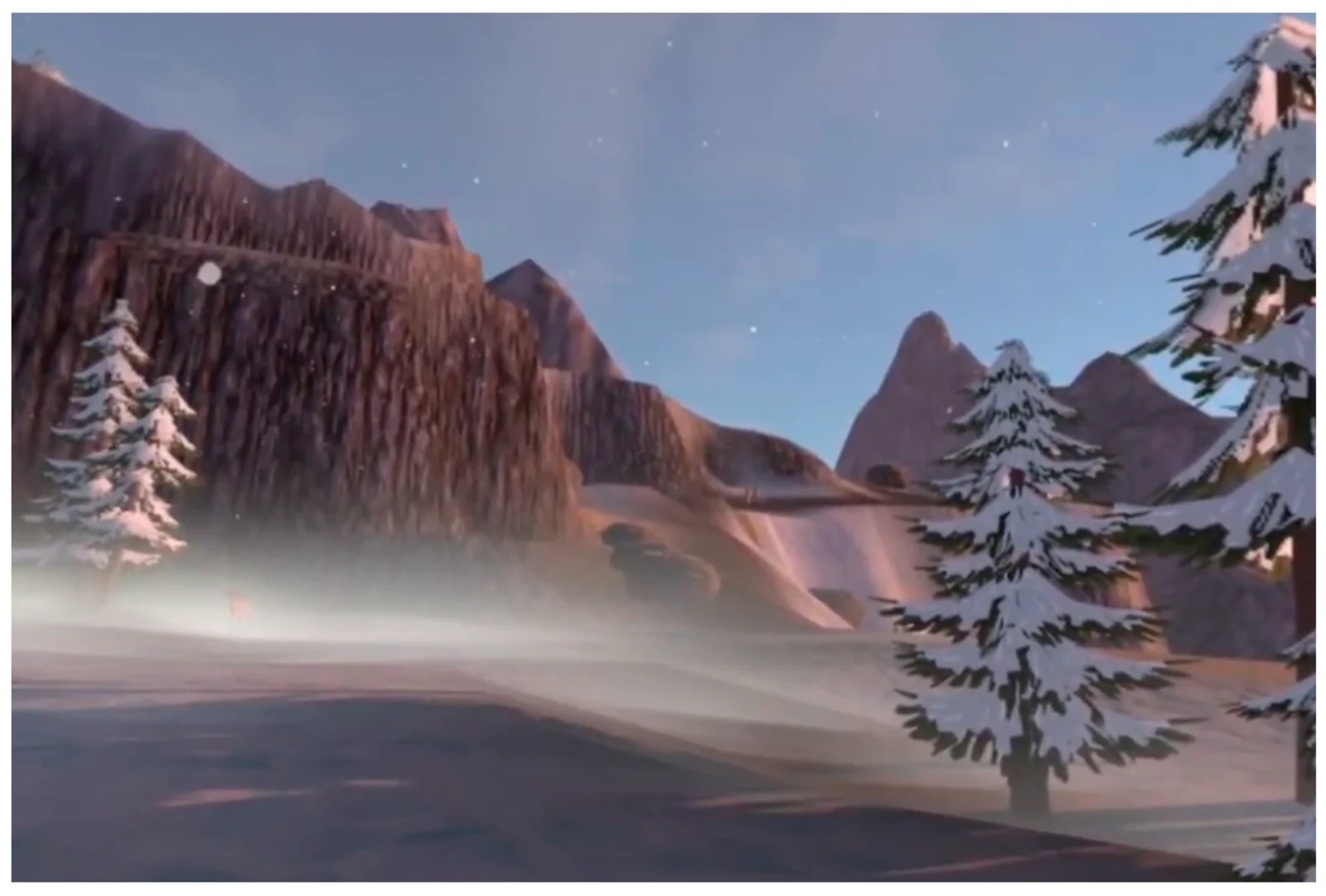
Scene 4.

**Figure A5:**
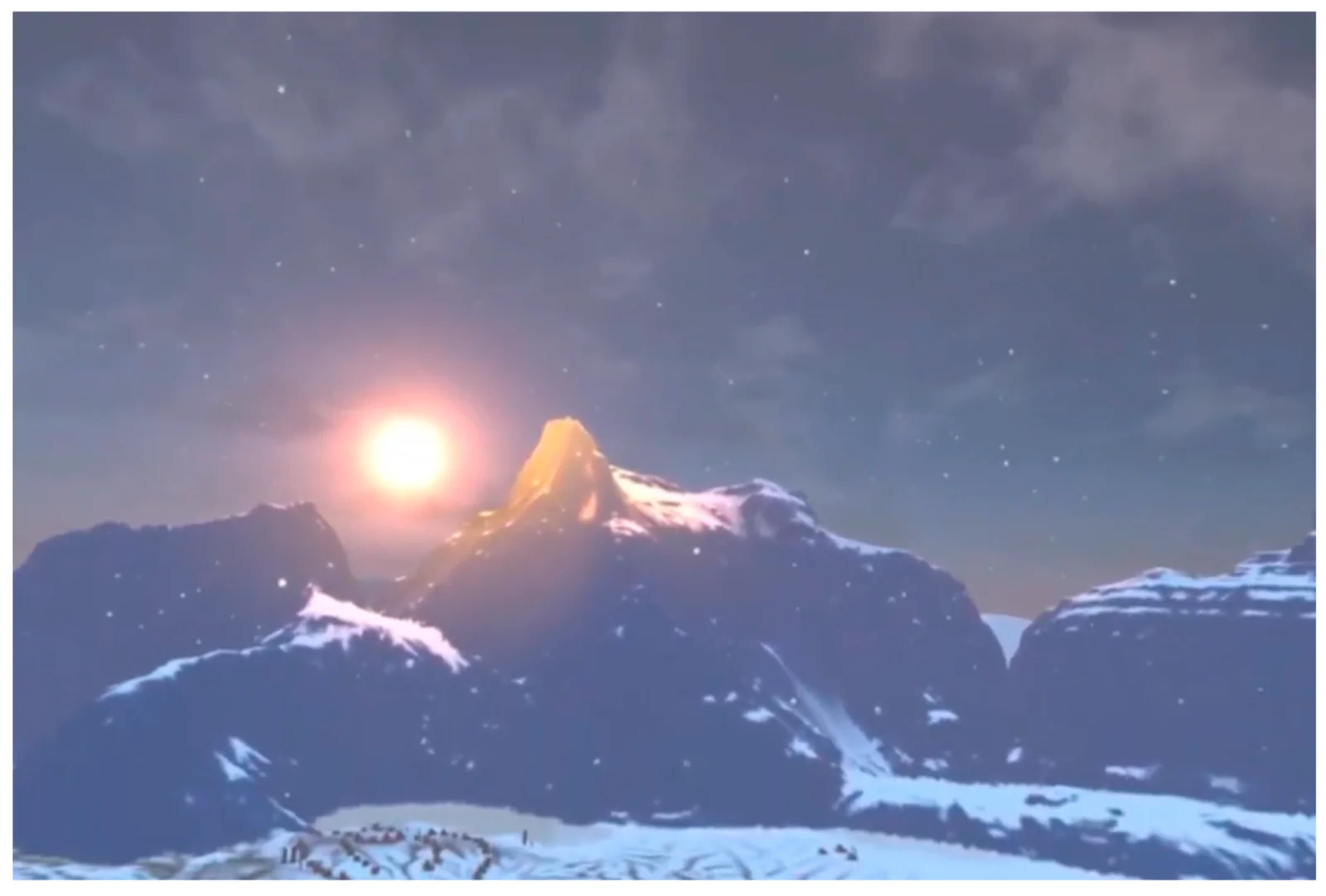
Scene 5.

**Figure A6:**
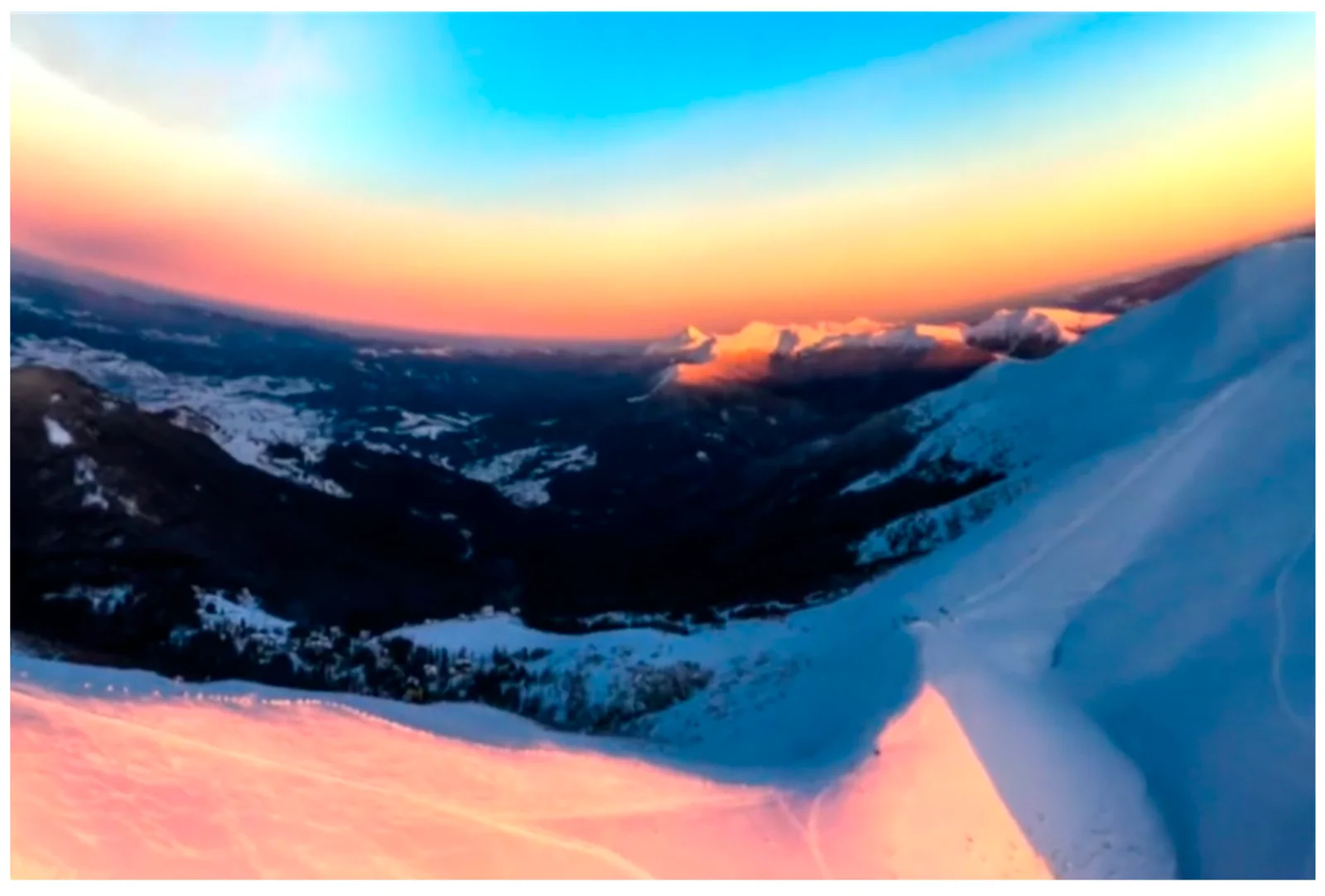
Scene 6.

**Figure A7:**
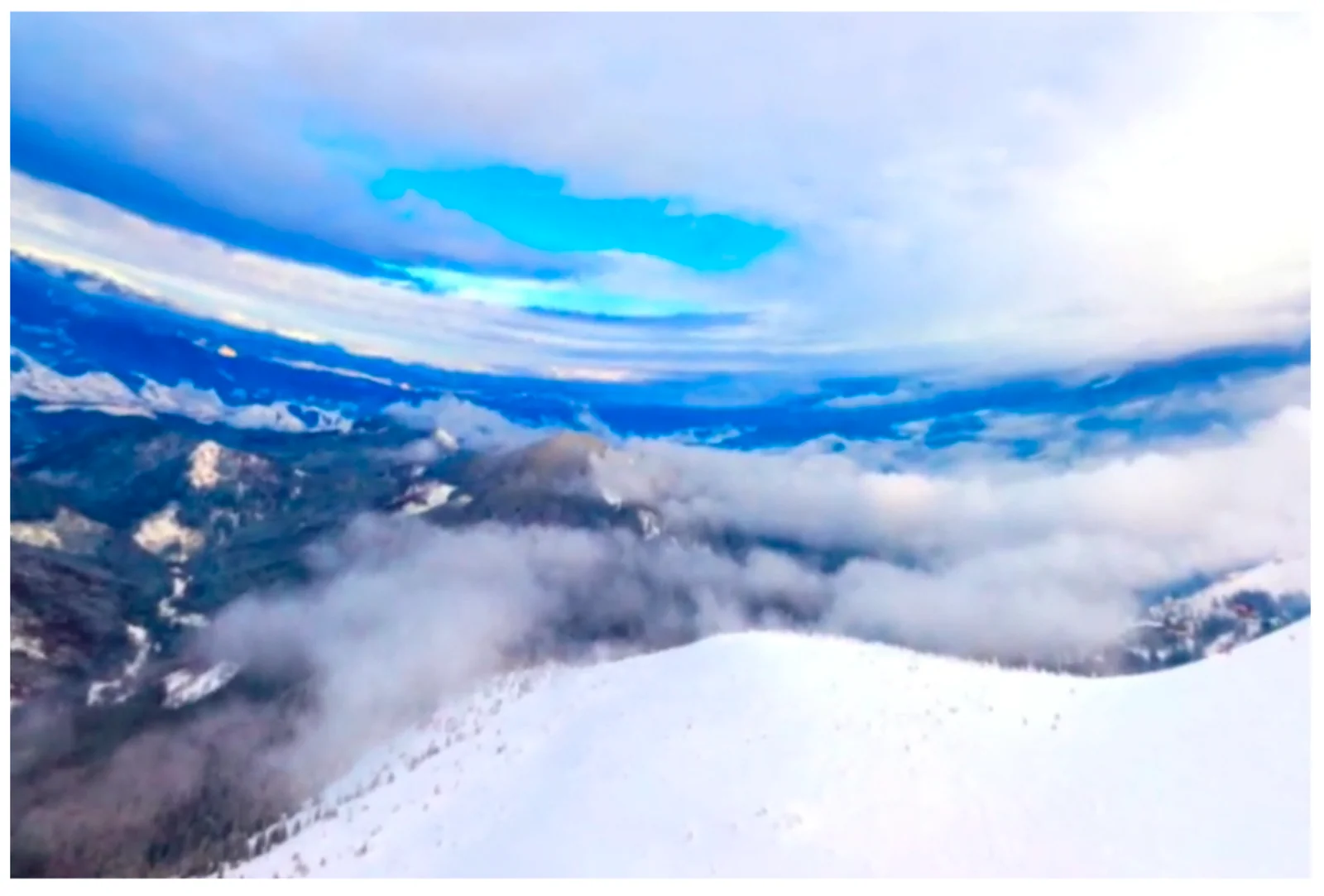
Scene 7.

## Data Availability

The data presented in this study are available on request from the senior author. The data are not publicly available due to privacy and ethical restrictions.

## Abbreviations

The following abbreviations are used in this manuscript:

AWE-S	Awe Experiences Scale
CLBP	chronic low back pain
HMD	head-mounted display
mDES	Modified Differential Emotions Scale
PANAS	Positive and Negative Affect Schedule
PHQ-9	Patient Health Questionnaire–9
PROMIS-Interference	PROMIS Pain Interference–Short Form 6b
SF-MPQ PRI	McGill Pain Questionnaire, Short Form: Pain Rating Index
TEI	Treatment Evaluation Inventory
VAS	Pain Visual Analogue Scale
VR	virtual reality

## Appendix B. Interview Guide

1. Can you describe your overall experience with virtual reality? What was it like for you?
2. Did you notice any changes in how you felt, or what you were thinking?
3. Did you notice any changes in your back pain?
4. What was it like emotionally for you through the virtual reality experience? Did you notice if you felt a certain way?
5. Did you notice any thoughts you had while you were in the VR? Or if your thoughts changed in any way?
6. Did anything in the VR experience particularly affect you? Any scenes? How was the music?
7. What would you have liked to see in virtual reality?
8. Anything you didn’t like, or that felt weird?
9. How was it coming out the virtual reality?
10. As we mentioned, we are working to incorporate these kinds of VR experiences into larger treatments for folks who have pain; do you have any thoughts about this or feedback you want to provide us?
11. If you had to rate your overall level of satisfaction regarding participating in the study, where 0 = extremely dissatisfied and 100 very satisfied, what rating would you provide?
12. Would you be interested in participating in this or a similar study again?
13. Is there anything else you want to tell us about your experiences in the study?
